# Exposure to arsenic and cognitive impairment in children: A systematic review

**DOI:** 10.1371/journal.pone.0319104

**Published:** 2025-02-26

**Authors:** Yumei Tian, Qi Hou, Mingyue Zhang, Er Gao, Yue Wu

**Affiliations:** 1 School of Nursing, Hunan Medical University, Huaihua City, Hunan Province, China; 2 Wuhan Polytechnic University, School of Life Sciences and Technology, Wuhan City, Hubei Province, China; 3 School of Nursing, Ningxia Medical University, Yinchuan City, Ningxia Province, China; University of Alcala Faculty of Medicine and Health Sciences: Universidad de Alcala Facultad de Medicina y Ciencias de la Salud, SPAIN

## Abstract

**Objective:**

Arsenic exposure is a significant public health concern, particularly for its impact on children’s cognitive development. Arsenic, a prevalent environmental toxin, is known to disrupt various biological pathways, leading to cognitive deficits and neurodevelopmental issues. Understanding the long-term effects and mechanisms underlying arsenic-induced cognitive impairments is crucial for devising effective interventions.

**Methods:**

This systematic review included observational and experimental studies focusing on children under 18 years exposed to arsenic through drinking water, food, or other environmental sources. Studies were selected through comprehensive database searches, encompassing articles that measured cognitive outcomes via standardized tests. The synthesis was primarily narrative, given the heterogeneity in study designs, exposure levels, and outcomes.

**Results:**

The review analysed findings from 24 studies, showing a consistent inverse relationship between arsenic exposure and cognitive performance in children. Higher arsenic levels were associated with lower IQ scores, slower processing speeds, and impaired memory and language skills. These cognitive deficits were evident across diverse geographical regions and persisted even after adjusting for sociodemographic factors. The studies highlighted the potential for both immediate and long-term cognitive effects, underscoring the importance of early-life exposure.

**Conclusions:**

Arsenic exposure has the potential to impair cognitive development in children. Nonetheless, quantitative meta-analysis is necessary to deduce any conclusions related to its impact. Public health efforts must prioritize reducing arsenic exposure through improved water quality and community-awareness programs. Future research should focus on longitudinal studies to better understand the dose-response relationship and the effectiveness of intervention strategies.

**Systematic review registration:**

Prospero, CRD42024544442.

## Introduction

Arsenic exposure is a significant global health concern, affecting millions of people worldwide [1]. One of the primary routes of exposure is through drinking water and food, particularly in regions where the contamination of groundwater and agricultural products is prevalent [2]. The toxic effects of arsenic have been widely studied, particularly concerning carcinogenic risks, cardiovascular issues, and neurotoxic impacts [3–5]. However, much of this research has centred on adult populations, leaving a critical gap in our understanding of the long-term effects of arsenic exposure on children [3–5].

Recent years have seen growing recognition of the unique vulnerabilities of children to environmental toxins. The developmental processes that occur from conception through adolescence are incredibly sensitive to chemical disruptions, and arsenic exposure during these periods can have lasting and potentially irreversible effects. In particular, there is mounting evidence suggesting that early-life exposure to arsenic can impair neurodevelopment, leading to cognitive deficits, learning disabilities, and other neurobehavioral issues [6–8].

One of the key ways in which arsenic can impact neurodevelopment is through its influence on cellular processes and brain structure [3,9]. Arsenic is known to interfere with various metabolic pathways, including those involved in neurotransmitter production and signalling [3,9]. This interference can disrupt the delicate balance required for healthy brain function, leading to deficits in cognitive performance. For example, arsenic exposure has been shown to reduce the activity of acetylcholinesterase, an enzyme critical for breaking down the neurotransmitter acetylcholine. This disruption can impair synaptic transmission, affecting learning and memory functions [3,9].

Beyond its immediate neurotoxic effects, arsenic can also induce oxidative stress and inflammation in the brain, further compounding its impact on neurodevelopment. Oxidative stress, characterized by an imbalance between free radicals and antioxidants in the body, can damage neurons and other brain cells [10]. Inflammatory responses can exacerbate this damage, leading to cellular apoptosis and potentially contributing to neurodegenerative conditions [9,10]. These mechanisms underscore the multifaceted nature of arsenic’s impact on cognitive development, highlighting the need for comprehensive research into its long-term effects on children’s cognitive health.

The evidence linking arsenic exposure to cognitive impairment in children comes from a variety of sources, including epidemiological studies, clinical research, and animal models [6–8]. Research from various other regions has explored the relationship between arsenic exposure and cognitive outcomes in children [6–8,11,12]. These studies have yielded mixed results, with some showing clear associations between higher arsenic levels and cognitive deficits, while others have found no significant effects [6–8,11,12]. This inconsistency may be due to differences in study design, sample size, exposure levels, or genetic and environmental factors that modulate susceptibility to arsenic’s neurotoxic effects. Nevertheless, the collective evidence points toward a concerning trend that warrants further investigation.

The implications of arsenic-induced cognitive impairment extend beyond individual health outcomes, with broader societal consequences. Cognitive deficits in childhood can hinder educational attainment, limit future employment opportunities, and contribute to socioeconomic disparities. Furthermore, the impacts of arsenic exposure on cognitive function may persist into adulthood, increasing the risk of neurodegenerative conditions such as Alzheimer’s disease or Parkinson’s disease.

In light of these concerns, there is an urgent need to examine the existing literature on arsenic exposure and cognitive impairment in children comprehensively. This study aims to address this need, synthesizing available evidence to provide a clearer understanding of the relationship between arsenic exposure and cognitive development.

## Methods

**Types of Studies:** Our review encompasses any form of observational studies (case-control, cohort, cross-sectional) and experimental trials that investigate the impact of arsenic exposure on cognitive outcomes in children. We include full-text articles and exclude publications available solely as abstracts or unpublished data.

**Population:** Children aged less than 18 years exposed to arsenic through drinking water, food, or other environmental sources.

**Exposures:** Measurement of arsenic exposure through either.

**Biological markers:** Including urinary or blood arsenic levels, or other biomarkers indicating recent or cumulative exposure.

**Environmental assessments:** Including water or soil arsenic levels or dietary arsenic intake.

### Outcomes of interest

**Cognitive Function:** in terms of IQ scores (full-scale, verbal, and performance) or scores from cognitive and neurobehavioral tests (e.g., processing speed, memory recall, and problem-solving skills).

### Search strategy

A comprehensive digital search strategy was implemented to identify relevant studies from various medical and scientific databases, including Medline, Scopus, Google Scholar, Cochrane library. A variety of key search terms were used in different combinations to refine the search, including “Arsenic Exposure,” “Cognitive Development,” “Neurobehavioral Outcomes,” “Arsenic Toxicity,” “Children’s Health,” and “Developmental Disorders.” This extensive search spanned from the inception of each database’s records to April 2024, without language restrictions to capture a wide range of international research findings.

### Study selection procedure

The study selection process was executed with meticulous precision by two independent reviewers. Initially, each reviewer conducted their own search, and then they jointly reviewed titles and abstracts to identify articles that might meet the inclusion criteria. The full texts of these preliminarily chosen studies were then carefully scrutinized by both reviewers, working independently, to verify their relevance against the predefined criteria. If disagreements arose, the reviewers sought to reach an agreement through discussion. In cases where consensus could not be achieved, a third reviewer was consulted.

### Data collection

Structured data extraction was employed under the supervision of the lead researcher. The information gathered included: study title, publication details, descriptions of study design, information about study population, research setting, sample size, baseline characteristics, and cognitive outcomes measured. We also extracted inclusion and exclusion criteria for participants, descriptions of exposure groups based on different levels of arsenic exposure (e.g., low, moderate, or high), duration of follow-up, and final outcomes such as cognitive test scores (IQ, memory, processing speed).

We ensured that all relevant data were extracted systematically using a predefined extraction form. In cases where key data points were not explicitly reported, efforts were made to interpret and include relevant findings based on available information in the studies. However, no studies were excluded from the review solely due to missing data, as our objective was to provide a comprehensive narrative synthesis. As no meta-analysis was conducted, missing data did not directly impact the analysis. Instead, any uncertainties or data gaps were clearly noted and considered in the interpretation of results.

### Risk of bias assessment

All the included studies were observational studies and hence, Newcastle-Ottawa Scale (NOS) was utilized for bias risk assessment [13]. We used the Newcastle-Ottawa Scale (NOS) to assess the risk of bias for the included studies. The NOS evaluates three broad perspectives: (1) Selection of the study groups, (2) Comparability of the groups, and (3) Outcome assessment. Selection criteria included aspects like the representativeness of the sample and ascertainment of exposure. Comparability was assessed based on control for key confounding factors, while outcome assessment focused on the robustness and adequacy of follow-up procedures. Each study is awarded a maximum of nine stars across these domains, with higher scores indicating better quality. Specifically, studies were classified as follows: scores of 7–9 were considered low risk of bias, scores of 4–6 were deemed moderate risk of bias, and scores of 0–3 indicated a high risk of bias.

### Data synthesis

Given the significant heterogeneity in participant profiles, exposure levels, and outcome measurements across the included studies, a narrative synthesis was employed in place of a meta-analysis. This approach enables a comprehensive qualitative evaluation of the findings, highlighting key relationships between arsenic exposure and cognitive impairment in children.

The narrative synthesis process was structured as follows:

### Categorization of studies

Studies were first categorized based on participant demographics, exposure levels, and the nature of the cognitive outcomes measured. This initial categorization allowed for a nuanced understanding of the different study contexts, laying the groundwork for a comparative analysis. This has been reported in Table 1.

**Table 1:** Characteristics and outcomes reported in included studies (N = 24).

Author	Country	Study design	Population	Sample size	Exposure Assessments	Method used	Outcome	Mean age	Gender distribution	Risk of bias assessment
Khan K at al. 2012	Bangladesh	School-based Cross-sectional study	Children at the age of 8 to 11 who had no known physical disability or chronic illness, and were not twins	840	119.5 (147.5) in water	Graphite furnace atomic absorption spectrophotometry (GFAA) using a Perkin-Elmer Analyst 600 system	Neither, WAs nor UAs was significantly related to mathematics achievement score and language test score (p < 0.05), with or without adjustment for other covariates. We did not observe associations between measures of Arsenic exposure and academic achievement.	Not available	Male – 396 (47.1) Female – 444 (52.9)	Low
Abbas S et al. 2012	Pakistan	Community based- Cross-sectional descriptive study	School going adult and children who lives in these three villages (Bangalacombo, Kamal chisti town, Qasar ghar) of Kasur city more than 5 years were included	Not available	In experimental group average concentration of arcenic in drinking water is 46% using Atomic absorption spectrophotometric and 44% using field kit.	Atomic absorption spectrophotometric and field kit.	In all the three sites the average height and weight of children is lower so is the intellectual functioning level, which shows the relationship of health status with their intellectual functioning level. This study shows that the intellectual functioning level of children drinking arsenic contaminated water significantly lower as compared to those, drinking arsenic-free water.	Not available	Not available	High
Calderon J et al. 2001	Mexico	Community based- Cross-sectional study	Children 6 to 9 years of age attending elementary schools	80	According to the CDC, 57.6% of the exposure zone children have values above the action level, while for reference zone children only 11.7% were above 50 lgAs/L in venous blood	Atomic absorption spectrophotometric method	Verbal comprehension, Longterm memory and attention are higher brain functions that could be affected in exposed children.	7.61 ± 0.8	Male – 41 (51.3) Female – 39 (48.7)	Moderate
Asadullah M N et al. 2011	Bangladesh	School-based Cohort study	School students from selected 321 schools of the union at 2005	7,710	Wells with more than 50 g of arsenic per litre were identified as contaminated	Not reported	Children with arsenic poisoned tubewell at home have around 0.07 standard deviations lower scores in secondary mathematics than their peers with safe tubewells. The effect is even a bigger negative, 0.20 standard deviations lower, in primary mathematics. There is a negative correlation between learning outcomes of children, and arsenic contamination of drinking water wells at home is causal.	13.1 ± 0.9	Not available	High
Ghosh S B et al. 2017	India	School based Cross-sectional study	Children studying in the 3rd and 4th standard at four selected primary schools, living more than 5 years in the locality and whose ages were in between 9 and 11 years were selected.	142	As (ppm) in of high-exposure group 0.0506 (0.01959) in water(	UV–Visible spectrophotometer (Sys-Tronics, UV–Vis double beam Spectro 1,203) with 1 cmquartz cell	Water quality of control group clearly revealed that mean arsenic levels is much lower compare to exposure group. On the other hand, mean IQ level is also higher than exposure group. Therefore, it can be reasonably suspected that arsenic is the main contributing factor for such low level of IQ among the school children in the study areas. The result of this study shows that WAs concentration was significantly associated with IQ scores determined by Raven’s Progressive matrices.	9.6 ± 2.3	Male – 66 (46.3) Female – 76 (53.7)	Low
Manju R et al. 2017	India	Community based- Cross-sectional study	Children between the ages 10 and 14 years who were born and brought up in the Hutti village were included	40	The ground water levels of arsenic in Hutti village was found to be 90 μg/L.	Not reported	This study indicates that exposure to arsenic in drinking water is associated with neurotoxic effects in children. The mean IQ grades of children were significantly lower in children chronically exposed to arsenic in the drinking water	12	Male – 13 (32.5) Female – 27 (67.5)	Low
Nahar M N et al. 2014	Bangladesh	School based Cross-sectional survey	Adolescents between 14 and 15 years old who were randomly selected from different villages in Sonargaon thana. The study groups were composed of individuals who had been exposed to As.	312	The mean As concentration in tube-well water was 71.7 lg/L and the mean urinary As concentration was 205.3 lg/L	Inductively coupled plasma mass spectrometry	The IQ in the high-[As]u group did not differ from that in the medium-[As]u group. The percent distributions of the IQ grades for the three [As]u groups (low, medium, and high; A very small percentage of the respondents from the high- [As]u group possessed above-average intellectual capacity, with most having average or below-average IQ grades.	Not available	Male - 138 (44.2) Female – 174 (55.8)	Moderate
Roy A et al 2011	Mexico	School based Cross-sectional study	Children 6–7 years old, living near a metal foundry in Torreo´n, Mexico, participated in the study	526	The median [interquartile range (IQR)] concentration of total urinary arsenic was 55.2 (39.7) mg/L, with a range of 7.7–215.9 mg/L. Approximately, 54% of the participating children had UAs concentra- tions above 50 mg/L.	Hydride generation atomic absorption spectrometry	compared with the lowest quartile of UAs, those in the second quartile received higher scores (β = 3.1, p < 0.05) on the Oppositional Behaviour rating. Those in the second and fourth quartile had a higher risk (OR = 2.1 and 2.0, respectively, po0.1) of receiving a score of 65 or above on the teacher ratings of Oppositional Behaviour	6.9 ± 0.4	Male – 292 (55.5) Female – 234 (44.5)	Moderate
Von Ehrensrein O S et al. 2007	India	Community based- Cross-sectional study	Children age 5 to 15 years who were selected from a source population of 7,683 people in West Bengal, India, in 2001–2003.	351	Arsenic concentrations in urine and peak water were slightly higher in boys (urine, mean SD 87 63 g/L; water, 157,355 g/L) than in girls (68 56 g/L, P 0.005; 136,279 g/L, P 0.5). Peak lifetime arsenic exposure in water was 0–9 g/L for 56% of children, 10–49 g/L for 10%, 50–99 g/L for 6%, and 100 g/L for 29%.	Flow injection analysis using atomic fluorescence detection	Increasing tertiles of urinary arsenic concentrations were associated with reductions in some of the children’s test scores. The strongest effects were seen in the vocabulary test. Effects were found for the vocabulary, picture completion, and object assembly tests with reductions between 12% and 20%, but the confidence intervals were broad.	Median age -9	Male – 190 (54) Female – 161 (46)	Moderate
Rocha-Amador D et al.	Mexico	Community based- Cross-sectional study	Children who had lived in the area since birth and who were from 6 to 10 years	132	Moct- ezuma (As 5.8 ± 1.3 μg/L); Salitral (As 169 ± 0.9 μg/L) and 5 de Fe- brero (As 194 ± 1.3 μg/L) in urine	Atomic Absorption Spectrophotometer with hydride system	Inverse associations between fluoride levels in urine and children’s Performance, Verbal, and Full IQ scores after adjusting for confounders (β values = −13.0, −15.6, −16.9, respectively; all p-values < 0.001). Arsenic levels in urine were inversely associated with Full IQ scores (β = −5.72, p = 0.003) after adjusting for confounders	8.3 ± 1.1	Not reported	High
Wang et al. 2007	China	Community based- Cross sectional study	Children between 8 and 12 years of age from Shanxi province, China	720	Medium-arsenic and high-arsenic groups had significantly elevated arsenic levels, with means of 142 μg/L and 190 μg/L, respectively in water.	Hydride generation atomic fluorescence spectrometry	Children’s IQ scores decreased from a mean of 105 in the control group to 101 in the medium-arsenic group, 101 in the high-fluoride group, and 95 in the high-arsenic group. The reductions in IQ scores in the medium-arsenic, high-fluoride, and high-arsenic groups compared to the control group were statistically significant.	9.0 ± 0.82	376/344	High
Wasserman et al.2004	Bangladesh	Community based- Cohort study	Randomly selected children of age 9.5 to 10.5	201	Low to extremely high concentrations up to 790 μg/L, with a mean of 117.8 μg/L across the 196 wells used by the children’s households.	Graphite furnace atomic absorption (GFAA)	Children with water arsenic levels > 50 μg/L achieved significantly lower Performance and Full-Scale scores compared to children with levels < 5.5 μg/L. The association was stronger for water arsenic levels than for urinary arsenic, possibly because some reduction in exposure had occurred between the well testing and child assessments.	10.0 ± 0.4	98/103	High
Wasserman et al 2014	Bangladesh	School based Cross sectional study	Children in grades 3–5 from three Maine school districts are included	272	The average well water arsenic concentration across the 272 children in the study was 9.88 μg/L.	High-resolution inductively-coupled plasma mass spectrometry (HR-ICP-MS).	Relatively low levels of arsenic exposure from household well water, at or above 5 μg/L, was associated with lower performance on intelligence tests among schoolchildren in Maine.	9.67 ± 1.18	145/127	High
Wasserman et al. 2007	Bangladesh	Community based- Cohort study	Randomly selected children of 5.75 years and 6.25 years of age whose parents participated in another prospective study of author	301	Arsenic concentrations in the tube well water ranged from 0.1 to 864 μg/L.The mean arsenic concentration was 120.1 μg/L	Graphite furnace atomic absorption (GFAA)	Higher levels of arsenic exposure from drinking water were associated with lower performance on tests of intellectual functioning, particularly non-verbal abilities.	6.1 ± 0.18	150/151	Moderate
Burgos et al.2017	Chile	Community based- Cross-sectional study	Participants were children aged 6–15 years old living in specific contaminated neighborhoods in Arica, with a total of 180 recruited through home visits and divided into exposure cohorts based on remediation timing and birth year from 1999 to 2003	180	Urine inorganic arsenic (μg/L) Median Cohort 1–20 (13–30) Cohort 2–17.5 (11–26) Cohort 3–20 (11–31)	Not reported	Evidence of poorer cognitive development and IQ scores among the children who were potentially exposed to higher levels of heavy metal contamination from the waste site early in life before it was remediated, compared to children born after remediation efforts.	pre-remediation cohort (mean age 13.5 years)during-remediation cohort (mean age 10 years), and youngest post-remediation cohort (mean age 7 years)	Not reported	Low
Hamadani et al. 2011	Bangladesh	Community based- Cohort study	Infants born between May 2002 and December 2003 were included.And were tested 7 months, 1.5 years and 5 years.	2,260	70% of the tube wells used for drinking water had arsenic concentrations above 10 μg/L, which was the Bangladesh drinking water standard at that time.	High-pressure liquid chromatography	Early life exposure to arsenic, particularly higher concurrent exposure at 5 years, was associated with lower verbal and full scale IQ scores specifically in girls, suggesting girls may be more vulnerable to arsenic-induced impairments in cognitive function during early childhood	5 years	1,175/1,085	Low
Desai et al. 2018	Uruguay	School based Cross-sectional study	All first-grade children who regularly attended the school from July 2009 and August 2013	353	The median UAs concentration was 11.9 μg/l (range = 1.4–93.9).	Exposure to As was measured based on the urinary concentration of iAs, MMA and DMA. HPLC-HG-ICP-MS (HG, hydride generation, selects inorganic arsenic and its methylated metabolites into the ICP-MS, Inductively Coupled Plasma Mass Spectrometry) were used to measure the concentration of As.	There was no independent association between urinary total arsenic and general cognitive abilities measured by the Woodcock-Muñoz tests.	6.75 years	195/158	Moderate
Wang et al.2022	China	Community based- cohort study	school-aged children from 2016 to 2017	148	The mean water As concentrations were 2, 3, 142, and 190 μg/L in the control, high-fluoride, medium-As, and high-As groups, respectively	Hydride generation atomic fluores- cence spectrometry (HG-AFS)	Prenatal low-level As exposure may negatively affect girls’ performance intelligence quotient (PIQ) and verbal intelligence quotient (VIQ).	89.90 ± 3.77 (months)	71/77	High
Zhou et al.2020	China	Community based- Cohort study	school-aged children from June 2009 to January 2010	296	0.5–193.09 μg/L in urine samples.	Inductively coupled plasma mass spectrometry (ICP-MS, NexION 300X, PerkinElmer, USA).	Arsenic did not show significant association with full intelligence quotient (IQ) [Beta coefficient = 1.120 ( − 0.738, 2.979)], verbal IQ [Beta coefficient = 0.155 ( − 1.676, 1.986)], or performance IQ [Beta coefficient = 1.992 ( − 0.082, 4.067)]	NR	170/126	High
Vaidya et al.2023	India	Community based- cohort study	participants aged 6 to 23 years from November 4, 2016, and May 4, 2019	1,014	9.65 (9.40) μg/L in urine.	Atomic absorption spectrometer with a flow injection hydride generator system (AA800; PerkinElmer) and Zeeman-effect background correction.	Sparse-partial least squares analysis was used to describe a negative association of arsenic exposure with executive function (r = − 0.12 [P = 5.4 × 10 − 4]), brain structure (r = − 0.20 [P = 1.8 × 10 − 8]), and functional connectivity (within network, r = − 0.12 [P = 7.5 × 10 − 4]; between network, r = − 0.23 [P = 1.8 × 10 − 10]).	14.86 [4.79]	589/425	Low
Rosado et al.2017	Mexico	School based Cross-sectional study	children 6–8 years of age	602	58.1 ± 33.2 µg/L in urine	Atomic Absorption Spectrophotometer	Linear and logistic regressions adjusted for hemoglobin concentration, PbB, and sociodemographic confounders showed a significant inverse association between UAs and Visual–Spatial Abilities with Figure Design, the Peabody Picture Vocabulary Test, the WISC-RM Digit Span subscale, Visual Search, and Letter Sequencing Tests (p < 0.05). Boys excreted significantly more UAs (p < 0.05) and were affected on different cognitive areas than girls.	83.4 ± 4.4 (months)	319/272	Moderate
Ossa et al.2023	Colombia	Community based- Cross-sectional study	Colombian children and adolescents from April to July 2019	70	1.96 ± 2.73 µg/L in blood.	atomic absorption spectrometry (Thermo Scientific model iCE 3,500) with a hydride generator (VP100)	Correlation analyses found, on the one hand, significant negative relationships in tests integrated within the verbal (CVB) and executive function (EF) components as well as evidence of positive relationships in the manifestation of errors in their performance with the levels of Hg and/or As transformed by biomarker (Log10).	12.2 ± 2.0	34/36	Moderate
Saxena et al.2022	Bangladesh	Community based- Cross-sectional study	adolescents aged 14–16 years from 2012 to 2016	572	4.8 ± (4.3) in blood	Perkin-Elmer NexION 350S equipped Elemental Scientific autosampler 4DX. ICP-MS-DRC	Linear regression revealed negative associations for Spatial Working Memory and both As and Mn (As B = −2.40, Mn B = −5.31, p < 0.05).	14.6 ± (0.7)	270/202	Moderate
Vahter et al.2020	Bangladesh	Community based- cohort study	children in a rural Bangladeshi area from May 2002 and December 2003	1,523	Median urinary arsenic at 10 years was 58 µg/L (range 7.3–940 µg/L)	Inductively coupled plasma mass spectro- metry (ICP-MS,	Multivariable-adjusted regression analysis showed that, compared to the first urinary arsenic quintile at 10 years ( < 30 µg/L), the third and fourth quintiles (30–45 and 46–73 µg/L, respectively) had 6–7 points lower Full developmental raw scores (B: − 7.23, 95% CI − 11.3; − 3.18, and B: − 6.37, 95% CI − 10.5; − 2.22, respectively), corresponding to ~ 0.2 SD. Verbal comprehension and Perceptual reasoning seemed to be affected.	9.51 ± 0.11	675/737	Low

### Examination of key findings

Studies were examined for key findings, including associations between arsenic exposure levels and specific cognitive outcomes, such as IQ scores. These findings were then synthesized to identify consistent patterns and trends.

### Integration of evidence

The synthesized findings were integrated into a comprehensive narrative, highlighting the overarching relationships between arsenic exposure and cognitive impairment in children. This narrative also considered potential confounding variables.

### Conclusions and recommendations

Finally, the narrative synthesis provided a conclusion summarizing the key findings and offering recommendations for both future research and policy interventions to address the cognitive risks associated with arsenic exposure in children. This is provided in the discussion section.

## Results

### Search results

**Fig 1:**
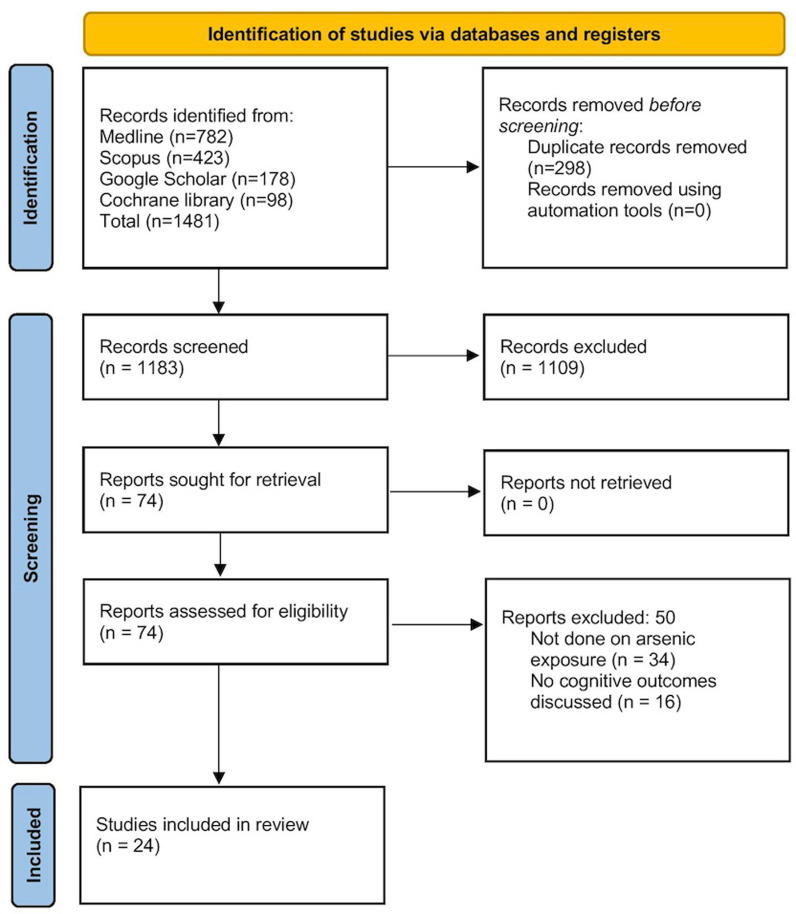
Systematic review flow chart.

The flowchart in Fig 1 outlines the process of identifying and selecting studies for a systematic review on arsenic exposure and cognitive outcomes. From an initial pool of 1,481 records found across multiple databases, 298 duplicates were removed and 1,109 records were excluded after screening. Overall, 74 studies were extracted for secondary screening out of which 50 were excluded (S1 Table). Ultimately, 24 studies met the eligibility criteria and were included in the review [6–8,11,12,14–32].

### Characteristics of the included studies

The 24 studies were conducted in various countries, including Bangladesh, India, Pakistan, Mexico, China, Uruguay, Brazil, Colombia, and Chile. Bangladesh featured prominently with several studies addressing the issue. The majority were cross-sectional studies, with a few cohort studies mixed in. The risk of bias was assessed as Low for seven studies and High for eight studies, while rest of the 9 studies were marked as Moderate, indicating variability in the quality of evidence. The studies varied widely in sample size, from as few as 40 participants to as many as 7,710. Children’s ages generally ranged from early childhood to late adolescence. Arsenic concentrations and exposure were reported differently across studies, with some using urine arsenic levels, others using water arsenic levels, and various methods to determine arsenic exposure. (Tables 1 and 2).

**Table 2:** Risk of bias assessment in the included studies (N = 24).

Author	Selection	Comparability	Outcome	Risk of bias assessment
Khan K at al. 2012	Low	Low	Low	Low
Abbas S et al. 2012	High	High	High	High
Calderon J et al. 2001	Low	Moderate	Low	Moderate
Asadullah M N et al. 2011	High	Low	High	High
Ghosh S B et al. 2017	Low	Low	Low	Low
Manju R et al. 2017	Low	Low	Low	Low
Nahar M N et al. 2014	Moderate	Moderate	Low	Moderate
Roy A et al 2011	Moderate	Low	Moderate	Moderate
Von Ehrensrein O S et al. 2007	Low	Moderate	Low	Moderate
Rocha-Amador D et al.	High	Low	High	High
Wang et al. 2007	High	High	High	High
Wasserman et al.2004	High	High	High	High
Wasserman et al 2014	Moderate	High	High	High
Wasserman et al. 2007	Low	Moderate	Low	Moderate
Burgos et al.2017	Low	Low	Low	Low
Hamadani et al. 2011	Low	Low	Moderate	Low
Desai et al. 2018	Low	Moderate	Low	Moderate
Wang et al.2022	High	Low	High	High
Zhou et al.2020	High	High	Low	High
Vaidya et al.2023	Low	Low	Low	Low
Rosado et al.2017	Low	Moderate	Low	Moderate
Ossa et al.2023	Moderate	Moderate	Low	Moderate
Saxena et al.2022	Moderate	Low	Moderate	Moderate
Vahter et al.2020	Low	Low	Low	Low

### Narrative synthesis findings

**Cognitive Deficits and IQ Scores:** Multiple studies have revealed a strong inverse association between arsenic exposure and children’s cognitive function. These studies show that children exposed to higher levels of arsenic have lower IQ scores, with the relationship persisting even after adjusting for sociodemographic factors.

#### Cognitive decline across various studies.

**Wasserman et al. (2004)** [31]: This study, conducted in Bangladesh, examined 201 children aged 10 years who had varying levels of arsenic exposure from drinking water. It found a significant inverse association between arsenic exposure and Full-Scale IQ, Performance IQ, and Verbal IQ scores, with the effect persisting after adjusting for sociodemographic covariates and other potential confounders. The study also noted a dose-response relationship, with children exposed to water arsenic levels exceeding 50 µg/L showing markedly lower Performance and Full-Scale IQ scores than those exposed to lower levels.

**Wang et al. (2007)** [11]: This study, conducted in Shanxi Province, China, examined 720 children aged 8–12 years and found a significant decline in IQ scores across groups with different arsenic exposures. The mean IQ scores decreased from 105 for the control group to 95 for the high-arsenic group, with the difference being statistically significant. The study also highlighted the need for remediation efforts to address arsenic exposure in the region.

**Rocha-Amador et al. (2007)** [19]: This study, conducted in Mexico, examined the relationship between arsenic exposure and cognitive function in 132 children aged 6–10 years across three rural communities with varying arsenic levels in drinking water. It found a significant inverse association between arsenic in drinking water and Full-Scale, Verbal, and Performance IQ scores, suggesting that arsenic exposure may contribute to cognitive impairment.

**Saxena et al. (2022)** [25]: This study examined the impact of arsenic exposure on children’s cognitive development in Bihar, India. The study found negative associations for spatial working memory and both arsenic and manganese levels.

**Zhou et al. (2020)** [30]: Conducted in China, this study examined the relationship between arsenic and other metals exposure and cognitive development in children. The study found that children exposed to higher arsenic levels did not show significantly difference in terms of full IQ, verbal IQ or performance IQ.

**Vaidya et al. (2023)** [26]: This recent study explored the cognitive effects of arsenic exposure in Nepalese children. The study found a negative association of arsenic exposure with executive function, brain structure, and functional connectivity, using sparse-partial least squares analysis. The study also underscored the importance of early interventions to reduce arsenic exposure, particularly in vulnerable populations.

**Ossa et al. (2023)** [27]: This study investigated the relationship between arsenic exposure and cognitive development in Colombian children. It found that children exposed to higher arsenic levels had lower cognitive test scores, particularly in language acquisition and problem-solving skills. The study also emphasized the importance of addressing arsenic contamination to protect children’s cognitive health.

**Calderon et al. (2001)** [22]: This study, conducted in Mexico, explored the neurodevelopmental effects of arsenic exposure on children aged 6–10 years living near a lead smelter. This study showed that the environmental exposure to arsenic influencing CNS function including verbal comprehension, long-term memory, and attention. This suggests that arsenic exposure, particularly from contaminated drinking water, can adversely affect specific cognitive domains.

**Manju et al. (2017)** [21]: This study examined the relationship between arsenic exposure and cognitive development in Indian children. Exposure to arsenic in drinking water was associated with neurotoxic effects and significantly lower mean IQ grades in children. The study also noted a dose-response relationship, with children exposed to higher arsenic levels showing greater cognitive impairments.

**Desai et al. (2018)** [7]: This study explored the impact of arsenic exposure on cognitive development in Pakistani children. The study found that there was no independent association between urinary total arsenic and general cognitive abilities.

**Vahter et al. (2020)** [29]: This study, conducted in Bangladesh, examined the relationship between arsenic exposure and neurodevelopmental outcomes in children. The study found that confirmed lower full developmental raw scores in higher arsenic quintiles, affecting verbal comprehension and perceptual reasoning. The study also noted the importance of controlling for confounding factors, such as socioeconomic status, to fully understand the relationship between arsenic exposure and cognitive impairment.

**Ghosh et al. (2017)** [20]: This study, conducted in Bangladesh, investigated the impact of arsenic exposure on cognitive development in children aged 8–12 years. The study found a significant negative association between arsenic exposure, as measured by drinking water levels, and IQ scores, with higher arsenic levels correlating with lower full-scale, verbal, and performance IQ scores as determined by Raven’s Progressive Matrices.

**Khan et al. (2012)** [24]: This study examined the relationship between arsenic exposure and cognitive development in Pakistani children. neither water arsenic (WAs) nor urinary arsenic (UAs) was significantly related to mathematics achievement score and language test score.

**Hamadani et al. (2011)** [18]: This study investigated the relationship between prenatal arsenic exposure and cognitive development in Bangladeshi children. Early life exposure to arsenic, especially at age 5, was associated with lower verbal and full scale IQ scores, specifically in girls. The study also highlighted the need for early interventions to reduce arsenic exposure, particularly during pregnancy.

**Asadullah et al. (2011)** [28]: This study examined the relationship between arsenic exposure and neurodevelopmental outcomes in Bangladeshi children. The study found a significant inverse association between exposure to arsenic-poisoned tubewells and lower scores in secondary and primary mathematics. The study also emphasized the importance of policy interventions to address arsenic exposure in contaminated regions.

**von Ehrenstein et al. (2007)** [16]: This study explored the relationship between arsenic exposure and cognitive development in Bangladeshi children. The study found a significant negative correlation showing that increasing tertiles of urinary arsenic were associated with reductions in certain test scores, with the strongest effects on vocabulary. The study also highlighted the potential for cumulative effects, noting that children with prolonged arsenic exposure experienced greater cognitive impairment.

**Rosado et al. (2007)** [14]: This study investigated the impact of arsenic exposure on cognitive development in Mexican children. The study found significant inverse relationship between urinary arsenic and visual-spatial abilities, affecting different cognitive tests like the Peabody Picture Vocabulary Test. The study also emphasized the importance of addressing arsenic contamination in vulnerable regions to protect children’s cognitive health.

**Abbas et al. (2012)** [6]: This study examined the relationship between arsenic exposure and neurodevelopmental outcomes in Pakistani children. The study found a significant inverse association between arsenic levels and intellectual functioning. The study also emphasized the need for remediation efforts to reduce arsenic exposure in contaminated regions.

**Nahar et al. (2014)** [12]: This study investigated the relationship between prenatal arsenic exposure and cognitive development in Bangladeshi children. The study found a significant inverse association with high arsenic concentration significantly lowering the mean IQ percentile, but the IQ in high arsenic groups did not differ significantly from medium groups. This highlights the potential for high arsenic exposure to contribute to cognitive deficits.

**Roy et al. (2011)** [15]: This study examined the impact of arsenic exposure on cognitive development in Bangladeshi children aged 8–12 years. The study found those in the second quartile of urinary arsenic had higher scores on oppositional behaviour ratings. The study also noted that arsenic exposure’s neurotoxic effects may contribute to deficits across multiple domains.

**Wasserman et al. (2007)** [32]: This study, conducted in Bangladesh, expanded upon earlier findings by exploring the relationship between arsenic exposure and intellectual function in 301 6-year-old children. It found a significant inverse association between arsenic levels in drinking water and cognitive test scores, particularly in areas such as performance IQ and processing speed. After controlling for various sociodemographic factors and water manganese levels, arsenic exposure remained negatively correlated with cognitive function, suggesting arsenic’s neurotoxic effects on children’s intellectual development.

**Wasserman et al. (2014)** [8]: This study investigated the long-term effects of arsenic exposure on cognitive development in Bangladeshi children, particularly those aged 10 years. The study found a significant inverse association between arsenic exposure and cognitive test scores across multiple domains, including full-scale IQ, verbal IQ, and performance IQ. These findings highlight arsenic’s enduring impact on cognitive development, emphasizing the need for early interventions to reduce arsenic exposure from drinking water.

**Burgos et al. (2017)** [17]: This study explored the impact of environmental arsenic exposure on cognitive development in Chilean children aged 6–15 years. The study found a significant inverse association between arsenic exposure and IQ scores, particularly in children from neighborhoods near waste disposal sites containing heavy metals. The study also found that children born after remediation efforts had significantly higher cognitive test scores than those born before, indicating the potential for mitigation measures to reduce arsenic’s neurotoxic effects.

**Wang et al. (2022)** [23]: This study examined the impact of arsenic exposure on cognitive development in Chinese children. The study has reported a significant association with prenatal low-level arsenic exposure negatively affected the girls’ performance intelligence quotient (PIQ) and verbal intelligence quotient (VIQ).

***Findings based on the Exposure Matrices***: Wasserman et al. (2004) primarily investigated arsenic exposure through drinking water, urine, and blood matrices. The study reported a significant inverse association between cognitive decline and arsenic levels in drinking water. Associations with arsenic levels in urine and blood were less pronounced, suggesting that water arsenic levels, likely reflecting direct and prolonged exposure, were the most robust predictors of cognitive outcomes. This highlights the differential significance of exposure matrices in interpreting the cognitive impacts of arsenic exposure.

Similarly, Wang et al. (2007) observed stronger associations between arsenic concentrations in drinking water and reduced IQ scores compared to urinary arsenic. These findings align with the hypothesis that drinking water serves as a primary exposure source, potentially leading to higher systemic absorption compared to other exposure matrices.

In contrast, Nahar et al. (2014) found that urinary arsenic concentrations were more predictive of cognitive deficits than water arsenic levels, indicating that urine, as a biomarker of recent arsenic exposure, may provide insights into the body’s acute response to arsenic exposure. Such findings underscore the importance of understanding the temporal and source-specific variations in arsenic exposure when interpreting its effects on cognitive development. Across the reviewed studies, it is evident that the choice of exposure matrix—be it drinking water, urine, or blood—has implications for the observed associations and their interpretation.

### Subdomain-specific deficits

#### Processing speed.

Arsenic exposure has been consistently associated with deficits in processing speed, a crucial component of cognitive function. Studies from various regions [15–18,20,23,29], including Bangladesh, China, and Mexico, have demonstrated an inverse relationship between arsenic exposure and processing speed scores in children. These findings suggest that arsenic may impair various aspects of neurodevelopment, leading to slower information processing and reaction times. Furthermore, these deficits persist even after adjusting for various confounding factors, indicating arsenic’s direct impact on cognitive function. The dose-response relationship observed in several studies further emphasizes that higher arsenic exposure correlates with more significant impairments in processing speed.

#### Language and communication.

Beyond overall IQ scores, arsenic exposure has also been linked to deficits in language acquisition and communication skills. Research indicates that children exposed to higher arsenic levels show lower verbal IQ scores and perform worse on language-related tests, affecting their ability to comprehend and produce language effectively [6,7,11,12,14–16,28,30,32]. This relationship is particularly concerning, as language acquisition and communication skills are foundational to cognitive development and learning. The compounded effect of these deficits can contribute to broader cognitive impairments, highlighting arsenic’s multifaceted neurotoxic impact.

#### Neurodevelopmental tests.

Children exposed to arsenic-contaminated water have shown deficits across various cognitive domains, including processing speed, verbal IQ, and performance IQ. Research indicates that these deficits are pervasive, with higher arsenic exposure correlating with lower cognitive test scores across the board [7,12,21–24,30–32]. The dose-response relationship observed in many studies underscores the significance of arsenic’s impact, with higher exposure levels leading to more substantial cognitive impairments. These findings highlight the need for comprehensive interventions to address arsenic contamination and protect children’s neurodevelopmental health.

#### Dose-response relationship.

Several studies demonstrate a clear dose-response relationship between arsenic exposure levels and cognitive outcomes in children [6,7,11,12,14,15,17,20,22–24,28–32]. This relationship indicates that higher arsenic levels correlate with more significant declines in intellectual function. The consistent observation of this dose-response relationship across diverse studies and regions highlights arsenic’s detrimental impact on cognitive development. The dose-response relationship also suggests that prolonged or higher levels of arsenic exposure can exacerbate cognitive deficits, including IQ scores and neurodevelopmental test results. This underscores the importance of addressing arsenic exposure early on to prevent significant cognitive impairment.

The review identified that dose-response relationships varied depending on the exposure levels, matrices, and health outcomes assessed. For example, Wasserman et al. (2004) reported a significant dose-response relationship between arsenic concentrations in drinking water (ranging from 10 µg/L to 150 µg/L) and declines in IQ scores among children, with stronger effects at higher exposure levels. In contrast, studies such as Nahar et al. (2014) highlighted dose-response relationships based on urinary arsenic concentrations, where levels above 50 µg/g creatinine were associated with a higher prevalence of cognitive deficits. Similarly, Wang et al. (2007) found a consistent dose-response pattern for arsenic concentrations in drinking water, with neurocognitive impairment increasing at exposure levels exceeding 100 µg/L.

The consistency of dose-response findings was influenced by the matrices used for exposure assessment. Drinking water arsenic concentrations demonstrated more robust and consistent dose-response relationships, likely due to their reflection of long-term cumulative exposure. Urinary arsenic levels, representing more recent exposure, showed variability across studies due to differences in temporal exposure and individual metabolism. This underscores the importance of considering the exposure matrix in interpreting dose-response relationships.

#### Neurotoxicity mechanisms.

Arsenic’s neurotoxic effects are linked to its disruption of multiple metabolic pathways. One key mechanism involves interfering with neurotransmitter production and signalling, which is essential for healthy brain function. This disruption can lead to impairments in various cognitive domains, including processing speed, language acquisition, and problem-solving skills. Additionally, arsenic exposure can induce oxidative stress and inflammation in the brain, leading to cellular damage. This damage can further contribute to cognitive deficits and neurodevelopmental impairment, emphasizing arsenic’s multifaceted impact on children’s cognitive health [6–8,11,12,14–32].

### Longitudinal impacts

#### Long-term effects.

While most studies included in this review are cross-sectional, they collectively hint at the potential long-term cognitive impacts of arsenic exposure. For instance, Hamadani et al. (2011) [18] examined cognitive function in children exposed to arsenic in utero and found that even early-life exposure could contribute to cognitive deficits later in life. This suggests that arsenic’s neurotoxic effects may persist over time, potentially leading to cumulative cognitive deficits. The longitudinal impacts of arsenic exposure underscore the importance of interventions to reduce exposure early on, particularly in vulnerable populations.

#### Cumulative exposure.

Several studies suggest that the cognitive deficits associated with arsenic exposure may be cumulative in nature, with children exposed to higher levels for longer periods experiencing more significant impairment [15–17,21,23,24,28,30]. This cumulative effect is particularly concerning, as it suggests that prolonged arsenic exposure can lead to lasting and potentially irreversible cognitive decline. The dose-response relationship observed in many studies reinforces this notion, with higher arsenic levels correlating with more substantial declines in IQ scores and other cognitive test results. This cumulative effect underscores the need for interventions to reduce arsenic exposure early in life to prevent long-term cognitive decline and protect children’s cognitive health.

### Regional and cultural differences

#### International consistency.

Studies from various regions, including Bangladesh, China, and Mexico, consistently show an inverse relationship between arsenic exposure and cognitive function, despite differing cultural and regional contexts [15–18,20,23,29]. This consistency across diverse populations strengthens the argument for arsenic’s neurotoxic effects on children’s cognitive development, highlighting its global impact. The consistent findings across various regions also suggest that arsenic’s neurotoxic effects are not confined to specific cultural or environmental factors, reinforcing the need for global remediation efforts to address arsenic contamination.

#### Socioeconomic factors.

Several studies account for socioeconomic factors, such as parental education and household income, highlighting that arsenic exposure remains a significant predictor of cognitive deficits even when these factors are controlled [6,7,11,12,14,16,20,22,24–26,30–32]. This indicates that the neurotoxic effects of arsenic are independent of socioeconomic status, emphasizing the need for interventions to reduce arsenic exposure across diverse populations. The consistent association between arsenic exposure and cognitive decline, even after accounting for socioeconomic factors, underscores the direct impact of arsenic on neurodevelopmental health.

#### Confounding factors.

Many studies included in this review have considered various confounding factors that could influence cognitive development in children, including socioeconomic status, parental education, and co-exposure to other toxins [11,14,16,20,22,23,25,28,30–32]. The findings reveal that while these factors do play a role in cognitive outcomes, arsenic exposure remains a significant predictor of cognitive impairment, even after controlling for these variables. For example, parental education and household income can influence children’s access to educational resources and their overall learning environment, yet studies show that arsenic exposure independently correlates with cognitive deficits. This underscores arsenic’s direct neurotoxic impact on children’s cognitive development.

## Discussion

The systematic review on the neurodevelopmental impact of arsenic exposure on children reveals a complex interplay between environmental factors and cognitive development, elucidating significant public health concerns. The pervasive nature of arsenic exposure, particularly in vulnerable regions, underscores an urgent need for targeted interventions and policy reforms.

Arsenic’s neurotoxicity, as evidenced in the included studies, manifests in several key areas of cognitive function, including processing speed, language acquisition, and problem-solving abilities [23–30]. These cognitive domains are crucial for the overall developmental trajectory of a child, affecting educational outcomes and future socioeconomic status. The findings consistently show that arsenic exposure correlates with lower IQ scores and impaired cognitive function, even after adjusting for confounding factors such as socioeconomic status and parental education [11,12,19–31].

The biologic mechanisms proposed for arsenic’s neurotoxic effects include disruption of neurotransmitter signalling, induction of oxidative stress, and promotion of inflammation within the brain [3,9]. These disruptions can lead to neuronal damage and apoptosis, which are likely contributors to the cognitive deficits observed. This mechanistic understanding is crucial as it not only helps in diagnosing the extent of damage but also in guiding therapeutic interventions aimed at mitigating the neurotoxic effects [3,9].

Further exploration into arsenic’s neurotoxic mechanisms reveals its potential to disrupt mitochondrial function, leading to decreased energy production within brain cells. This mitochondrial dysfunction can exacerbate the production of reactive oxygen species, thus amplifying oxidative stress and promoting a cycle of cellular damage and death. Additionally, arsenic interferes with the developmental regulation of gene expression in neural cells, which can alter the normal patterns of brain development and lead to structural and functional abnormalities. These insights into the cellular and molecular effects of arsenic provide a clearer picture of how extensive the damage can be, emphasizing the need for protective measures during critical periods of brain development [3,9].

Examining regional variations in the impact of arsenic exposure also reveals significant cultural considerations that influence both exposure levels and outcomes. Dietary habits, local water sourcing practices, and traditional medicine use can all affect arsenic exposure. For instance, regions with a high dependence on groundwater for drinking and irrigation may experience higher exposure levels, influencing the degree of cognitive impairments observed in these populations [33]. Cultural factors also play a crucial role in the acceptance and success of intervention strategies. Tailoring interventions to fit local customs and practices can enhance their effectiveness, promoting better compliance and more profound health impacts. This calls for culturally sensitive approaches to designing and implementing arsenic mitigation strategies, ensuring they are not only scientifically sound but also socially acceptable.

The variability in methods used for arsenic detection across the included studies contributes significantly to differences in sensitivity, specificity, and detection limits. Among the methods reported, Inductively Coupled Plasma Mass Spectrometry (ICP-MS) offers the highest sensitivity with detection limits as low as 0.0003 ppb, whereas X-Ray Fluorescence (XRF) and Anion Exchange High-Performance Liquid Chromatography (AE-HPLC) have higher limits of detection at approximately 0.7 ppb and 0.2 to 0.8 ppb, respectively. This methodological heterogeneity partly explains the discrepancies observed in arsenic concentration levels reported in different studies, especially in regions where concentrations are close to the detection limits of less sensitive methods. For instance, studies employing ICP-MS reported lower detectable arsenic levels compared to studies using XRF, which could influence risk assessments and mitigation strategies. Furthermore, the choice of method impacts the reliability of arsenic speciation, as methods like AE-HPLC are more suited for distinguishing between arsenite and arsenate compared to ICP-MS, which primarily quantifies total arsenic. These differences underscore the importance of considering detection methods when interpreting and comparing results, as well as when designing future studies to standardize analytical approaches and minimize variability.

The global nature of arsenic exposure and its profound impact on child health necessitates a coordinated response from public health authorities and governments. Interventions need to focus on preventing exposure, especially in high-risk areas, by improving water quality and establishing strict regulations on arsenic levels in consumer products and industrial emissions. The studies highlight the effectiveness of community-based interventions, such as the installation of arsenic-removal systems, which have led to improved cognitive outcomes in children from previously high-exposure areas [34].

Moreover, the evidence suggests that early-life exposure to arsenic could have lasting effects that extend into adulthood, potentially increasing the risk of neurodegenerative diseases. This long-term risk reinforces the need for early and aggressive intervention strategies that not only address immediate exposure but also monitor populations over time for emerging cognitive issues.

The dose-response relationships identified in this review reveal important insights into the health effects of arsenic exposure. The observed associations between arsenic exposure and adverse outcomes, such as cognitive decline, were strongly influenced by the exposure matrices and assessment methods used. Studies measuring arsenic levels in drinking water, such as Wasserman et al. (2004) and Wang et al. (2007), demonstrated more consistent dose-response relationships, particularly at exposure levels exceeding 100 µg/L. This consistency likely stems from drinking water’s role as a primary and cumulative source of arsenic exposure, providing a clearer link to long-term health effects.

In contrast, studies using urinary arsenic levels, such as Nahar et al. (2014), showed dose-response relationships that varied based on recent exposure and individual differences in arsenic metabolism. These variations underscore the temporal nature of urinary arsenic as a biomarker and its potential to capture short-term exposure rather than cumulative effects. Blood arsenic levels, used less frequently, were often intermediate in terms of reflecting both recent and long-term exposure, contributing to inconsistencies in dose-response findings.

The variability in dose-response relationships across studies highlights the critical role of exposure assessment methods and matrices. While drinking water arsenic concentrations provided the most consistent results, urinary and blood arsenic measurements introduced variability due to differences in individual metabolism, exposure timing, and environmental factors. These findings emphasize the need for future studies to standardize exposure assessment methods and consider the matrix’s influence when interpreting dose-response relationships.

The socioeconomic impact of cognitive deficits induced by arsenic exposure cannot be overstated. Children suffering from these deficits are likely to face challenges in academic achievement, resulting in lower educational attainment and reduced job prospects in adulthood [10,18]. This perpetuates a cycle of poverty in communities already vulnerable to environmental toxins. Furthermore, the burden of cognitive deficits extends beyond the individual to the societal level, where increased healthcare costs and lost productivity impose significant economic strains. Addressing these socioeconomic impacts requires integrated strategies that combine health interventions with educational support programs, ensuring that affected children can achieve optimal developmental outcomes despite their exposure to arsenic.

Some studies have explored the impact of other contaminants alongside arsenic, particularly fluoride, to understand how multiple environmental toxins interact and affect cognitive development. These studies consistently find that arsenic exposure correlates with cognitive decline, even when accounting for the influence of other toxins [11,12,23,26,30,32]. For instance, research conducted in China and Bangladesh has shown that arsenic exposure’s neurotoxic effects persist across various cognitive domains, including IQ scores, processing speed, and memory recall, despite the presence of other contaminants like fluoride. This highlights the need for comprehensive interventions to address arsenic exposure specifically and reduce its direct impact on children’s cognitive health.

Despite the extensive data linking arsenic exposure to cognitive deficits, there remain significant gaps in our understanding of the dose-response relationship and the potential for recovery following exposure cessation. Future research should aim to define the threshold levels of arsenic exposure that lead to detectable cognitive impairments and to evaluate the effectiveness of various mitigation strategies in reversing these effects.

Additionally, there is a need for longitudinal studies that follow children over longer periods to assess the lasting impacts of early-life exposure and the efficacy of early interventions. Such studies would provide invaluable insights into the critical windows of vulnerability and the potential for neuroplasticity and recovery in the developing brain.

This study has several notable strengths. First, it offers a comprehensive analysis of existing data on the impact of arsenic exposure on cognitive functions, synthesizing a wide range of sources. The use of established risk assessment tools such as the Newcastle-Ottawa Scale ensures a structured evaluation of study quality. Additionally, a rigorous data extraction and analysis protocol enhances the reliability of the results.

However, some limitations should also be acknowledged. The included studies varied significantly in terms of population, dosage, and duration of exposure, leading to considerable heterogeneity in the findings. This variation may limit the generalizability of the results to broader populations. Moreover, most of the included studies were observational, which restricts our ability to establish causality between arsenic exposure and memory impairment. Lastly, despite efforts to gather all relevant studies, potential publication bias cannot be completely ruled out.

## Conclusion

The evidence from this systematic review clearly indicates that arsenic exposure is a significant risk factor for cognitive impairment in children, with potential long-term effects that could span a lifetime. The findings call for an immediate and robust response to reduce arsenic exposure in affected regions and to protect future generations from its debilitating effects on brain development and cognitive function. Policymakers, researchers, and public health officials must prioritize this issue, ensuring that children in all regions have the opportunity to achieve their full cognitive potential in a safe and supportive environment.

## Supporting information

S1 Table List of all the extracted studies for secondary screening (N = 74). (DOCX)

S1 File PRISMA 2020 Checklist. (DOCX)

S2 File Data file. (XLSX)
