# Supplementary material for: Exposure to arsenic and cognitive impairment in children: A systematic review
Source: PLoS One. 2025 Feb 26;20(2):e0319104. doi: 10.1371/journal.pone.0319104 (PMC11864541; doi:10.1371/journal.pone.0319104)
Supplement: S1 Table — (DOCX) [file pone.0319104.s001.docx]

**S1 Table: List of all the extracted studies for secondary screening (N=74)**

| **Reference** | **Included/ Excluded Decision** | **Reasons for exclusion (if excluded)** |
| --- | --- | --- |
| Abbas S, Mehmood E, Qureshi A, Ahmad F, Vehra S, Khan A. An Assessment of Relationship Between Arsenic in Drinking Water, Health Status and Intellectual Functioning of Children in District Kasur. Pakistan Journal of Nutrition. 2012;11. doi:10.3923/pjn.2012.150.153 | Included | NA |
| Desai G, Barg G, Queirolo EI, Vahter M, Peregalli F, Mañay N, et al. A cross-sectional study of general cognitive abilities among Uruguayan school children with low-level arsenic exposure, potential effect modification by methylation capacity and dietary folate. Environ Res. 2018;164: 124–131. doi:10.1016/j.envres.2018.02.021 | Included | NA |
| Wasserman GA, Liu X, Loiacono NJ, Kline J, Factor-Litvak P, van Geen A, et al. A cross-sectional study of well water arsenic and child IQ in Maine schoolchildren. Environ Health. 2014;13: 23. doi:10.1186/1476-069X-13-23 | Included | NA |
| Wang S-X, Wang Z-H, Cheng X-T, Li J, Sang Z-P, Zhang X-D, et al. Arsenic and fluoride exposure in drinking water: children’s IQ and growth in Shanyin county, Shanxi province, China. Environ Health Perspect. 2007;115: 643–647. doi:10.1289/ehp.9270 | Included | NA |
| Nahar MN, Inaoka T, Fujimura M, Watanabe C, Shimizu H, Tasmin S, et al. Arsenic contamination in groundwater and its effects on adolescent intelligence and social competence in Bangladesh with special reference to daily drinking/cooking water intake. Environ Health Prev Med. 2014;19: 151–158. doi:10.1007/s12199-013-0369-z | Included | NA |
| Rosado JL, Ronquillo D, Kordas K, Rojas O, Alatorre J, Lopez P, et al. Arsenic exposure and cognitive performance in Mexican schoolchildren. Environ Health Perspect. 2007;115: 1371–1375. doi:10.1289/ehp.9961 | Included | NA |
| Roy A, Kordas K, Lopez P, Rosado JL, Cebrian ME, Vargas GG, et al. Association between arsenic exposure and behavior among first-graders from Torreón, Mexico. Environ Res. 2011;111: 670–676. doi:10.1016/j.envres.2011.03.003 | Included | NA |
| von Ehrenstein OS, Poddar S, Yuan Y, Mazumder DG, Eskenazi B, Basu A, et al. Children’s intellectual function in relation to arsenic exposure. Epidemiology. 2007;18: 44–51. doi:10.1097/01.ede.0000248900.65613.a9 | Included | NA |
| Burgos S, Tenorio M, Zapata P, Cáceres DD, Klarian J, Alvarez N, et al. Cognitive performance among cohorts of children exposed to a waste disposal site containing heavy metals in Chile. Int J Environ Health Res. 2017;27: 117–125. doi:10.1080/09603123.2017.1292494 | Included | NA |
| Hamadani JD, Tofail F, Nermell B, Gardner R, Shiraji S, Bottai M, et al. Critical windows of exposure for arsenic-associated impairment of cognitive function in pre-school girls and boys: a population-based cohort study. Int J Epidemiol. 2011;40: 1593–1604. doi:10.1093/ije/dyr176 | Included | NA |
| Rocha-Amador D, Navarro ME, Carrizales L, Morales R, Calderón J. Decreased intelligence in children and exposure to fluoride and arsenic in drinking water. Cad Saude Publica. 2007;23 Suppl 4: S579-587. doi:10.1590/s0102-311x2007001600018 | Included | NA |
| Ghosh S, Chakraborty D, Mondal N. Effect of Arsenic and Manganese Exposure on Intellectual Function of Children in Arsenic Stress Area of Purbasthali, Burdwan, West Bengal. Exposure and Health. 2017;9: 1–11. doi:10.1007/s12403-016-0216-8 | Included | NA |
| Manju R, Hegde AM, Parlees P, Keshan A. Environmental Arsenic Contamination and Its Effect on Intelligence Quotient of School Children in a Historic Gold Mining Area Hutti, North Karnataka, India: A Pilot Study. J Neurosci Rural Pract. 2017;8: 364–367. doi:10.4103/jnrp.jnrp_501_16 | Included | NA |
| Calderón J, Navarro ME, Jimenez-Capdeville ME, Santos-Diaz MA, Golden A, Rodriguez-Leyva I, et al. Exposure to arsenic and lead and neuropsychological development in Mexican children. Environ Res. 2001;85: 69–76. doi:10.1006/enrs.2000.4106 | Included | NA |
| Wang Y, Wang Y, Yan C. Gender differences in trace element exposures with cognitive abilities of school-aged children: a cohort study in Wujiang city, China. Environ Sci Pollut Res Int. 2022;29: 64807–64821. doi:10.1007/s11356-022-20353-4 | Included | NA |
| Khan K, Wasserman GA, Liu X, Ahmed E, Parvez F, Slavkovich V, et al. Manganese exposure from drinking water and children’s academic achievement. Neurotoxicology. 2012;33: 91–97. doi:10.1016/j.neuro.2011.12.002 | Included | NA |
| Saxena R, Gamble M, Wasserman GA, Liu X, Parvez F, Navas-Acien A, et al. Mixed metals exposure and cognitive function in Bangladeshi adolescents. Ecotoxicol Environ Saf. 2022;232: 113229. doi:10.1016/j.ecoenv.2022.113229 | Included | NA |
| Vaidya N, Holla B, Heron J, Sharma E, Zhang Y, Fernandes G, et al. Neurocognitive Analysis of Low-level Arsenic Exposure and Executive Function Mediated by Brain Anomalies Among Children, Adolescents, and Young Adults in India. JAMA Netw Open. 2023;6: e2312810. doi:10.1001/jamanetworkopen.2023.12810 | Included | NA |
| De la Ossa CA, Ramírez-Giraldo AF, Arroyo-Alvis K, Marrugo-Negrete J, Díez S. Neuropsychological effects and cognitive deficits associated with exposure to mercury and arsenic in children and adolescents of the Mojana region, Colombia. Environ Res. 2023;216: 114467. doi:10.1016/j.envres.2022.114467 | Included | NA |
| Asadullah MN, Chaudhury N. Poisoning the mind: Arsenic contamination of drinking water wells and children’s educational achievement in rural Bangladesh. Economics of Education Review. 2011;30: 873–888. doi:10.1016/j.econedurev.2011.05.001 | Included | NA |
| Vahter M, Skröder H, Rahman SM, Levi M, Derakhshani Hamadani J, Kippler M. Prenatal and childhood arsenic exposure through drinking water and food and cognitive abilities at 10 years of age: A prospective cohort study. Environ Int. 2020;139: 105723. doi:10.1016/j.envint.2020.105723 | Included | NA |
| Zhou T, Guo J, Zhang J, Xiao H, Qi X, Wu C, et al. Sex-Specific Differences in Cognitive Abilities Associated with Childhood Cadmium and Manganese Exposures in School-Age Children: a Prospective Cohort Study. Biol Trace Elem Res. 2020;193: 89–99. doi:10.1007/s12011-019-01703-9 | Included | NA |
| Wasserman GA, Liu X, Parvez F, Ahsan H, Factor-Litvak P, van Geen A, et al. Water arsenic exposure and children’s intellectual function in Araihazar, Bangladesh. Environ Health Perspect. 2004;112: 1329–1333. doi:10.1289/ehp.6964 | Included | NA |
| Wasserman GA, Liu X, Parvez F, Ahsan H, Factor-Litvak P, Kline J, et al. Water arsenic exposure and intellectual function in 6-year-old children in Araihazar, Bangladesh. Environ Health Perspect. 2007;115: 285–289. doi:10.1289/ehp.9501 | Included | NA |
| Mobile Media and Young Children's Cognitive Skills: A Review | Excluded | Not done on arsenic exposure |
| Cognitive Development | Excluded | Not done on arsenic exposure |
| Parental cognitive stimulation in preterm-born children's neurocognitive functioning during the preschool years: a systematic review | Excluded | Not done on arsenic exposure |
| Children's Cognitive Functioning in Disasters and Terrorism | Excluded | Not done on arsenic exposure |
| Fluorosis and cognitive development among children (6-14 years of age) in the endemic areas of the world: a review and critical analysis | Excluded | Not done on arsenic exposure |
| Cognitive assessment of refugee children: Effects of trauma and new language acquisition | Excluded | Not done on arsenic exposure |
| Acute exercise and children's cognitive functioning: What is the optimal dose of cognitive challenge? | Excluded | Not done on arsenic exposure |
| Cognitive development: children's knowledge about the mind | Excluded | Not done on arsenic exposure |
| Cognitive predictors of language abilities in primary school children: A cascaded developmental view | Excluded | Not done on arsenic exposure |
| Cognitive and academic growth among emergent bilingual children at risk and not at risk for math difficulties | Excluded | Not done on arsenic exposure |
| Socially Stratified Epigenetic Profiles Are Associated With Cognitive Functioning in Children and Adolescents | Excluded | Not done on arsenic exposure |
| Father- and Mother-Child Reminiscing About Past Pain and Young Children's Cognitive Skills | Excluded | Not done on arsenic exposure |
| Cognitive consistency and math-gender stereotypes in Singaporean children | Excluded | Not done on arsenic exposure |
| Scaffolding: Integrating social and cognitive perspectives on children's learning at home | Excluded | Not done on arsenic exposure |
| Cognitive reflection and authoritarianism relate to how parents respond to children's science questions | Excluded | Not done on arsenic exposure |
| Maternal depressive symptoms and children's cognitive development: Does early childcare and child's sex matter? | Excluded | Not done on arsenic exposure |
| Could it? Should it? Cognitive reflection facilitates children's reasoning about possibility and permissibility | Excluded | Not done on arsenic exposure |
| Inconsistencies between Subjective Reports of Cognitive Difficulties and Performance on Cognitive Tests are Associated with Elevated Internalising and Externalising Symptoms in Children with Learning-related Problems | Excluded | Not done on arsenic exposure |
| Young children's ability to report on past, future, and hypothetical pain states: a cognitive-developmental perspective | Excluded | Not done on arsenic exposure |
| Effects of Cognitive Reappraisal on Subjective and Neural Reactivity to Angry Faces in Children with Social Anxiety Disorder, Clinical Controls with Mixed Anxiety Disorders and Healthy Children | Excluded | Not done on arsenic exposure |
| Chronic Aflatoxin Exposure and Cognitive and Language Development in Young Children of Bangladesh: A Longitudinal Study | Excluded | Not done on arsenic exposure |
| Effects of maternal cigarette smoking during pregnancy on cognitive parameters of children and young adults: a literature review | Excluded | Not done on arsenic exposure |
| Creativity and flexibility in young children's use of external cognitive strategies | Excluded | Not done on arsenic exposure |
| Cognitive Function, Coping, and Depressive Symptoms in Children and Adolescents with Sickle Cell Disease | Excluded | Not done on arsenic exposure |
| Nutritional status and psychosocial stimulation associated with cognitive development in preschool children: A cross-sectional study at Western Terai, Nepal | Excluded | Not done on arsenic exposure |
| Children's family income is associated with cognitive function and volume of anterior not posterior hippocampus | Excluded | Not done on arsenic exposure |
| Environmental exposure to organophosphate pesticides and effects on cognitive functions in elementary school children in a Middle Eastern area | Excluded | Not done on arsenic exposure |
| Cognitive Performance in Adolescence: Links With Early Maternal Stimulation and Children's Anxious Behaviors | Excluded | Not done on arsenic exposure |
| Effects of Cognitively Engaging Physical Activity on Preschool Children's Cognitive Outcomes | Excluded | Not done on arsenic exposure |
| The development of children's understanding of death: cognitive and psychodynamic considerations | Excluded | Not done on arsenic exposure |
| Predicting children's emerging understanding of numbers | Excluded | Not done on arsenic exposure |
| Neuro-physiological correlates of sluggish cognitive tempo (SCT) symptoms in school-aged children | Excluded | Not done on arsenic exposure |
| Assessing children's cognitive flexibility with the Shape Trail Test | Excluded | Not done on arsenic exposure |
| Mobile Media and Young Children's Cognitive Skills: A Review | Excluded | Not done on arsenic exposure |
| Arsenic Metabolism in Children Differs From That in Adults | Excluded | No cognitive outcomes discussed |
| Genetic Susceptibility to Neurotoxicity Related to Prenatal Inorganic Arsenic Exposure in Young Spanish Children | Excluded | No cognitive outcomes discussed |
| Prenatal arsenic exposure, arsenic metabolism and neurocognitive development of 2-year-old children in low-arsenic areas | Excluded | No cognitive outcomes discussed |
| Low-level arsenic exposure and developmental neurotoxicity in children: A systematic review and risk assessment | Excluded | No cognitive outcomes discussed |
| Prenatal arsenic exposure, arsenic methylation efficiency, and neuropsychological development among preschool children in a Spanish birth cohort | Excluded | No cognitive outcomes discussed |
| Targeting the 'DNA methylation mark': Analysis of early epigenetic-alterations in children chronically exposed to arsenic | Excluded | No cognitive outcomes discussed |
| Long-term outcome of children with acute promyelocytic leukemia: a randomized study of oral versus intravenous arsenic by SCCLG-APL group | Excluded | No cognitive outcomes discussed |
| Arsenic on the hands of children after playing in playgrounds | Excluded | No cognitive outcomes discussed |
| The comparison of plasma arsenic concentration and urinary arsenic excretion during treatment with Realgar-Indigo naturalis formula and arsenic trioxide in children with acute promyelocytic leukemia | Excluded | No cognitive outcomes discussed |
| Contribution of diet and other factors for urinary concentrations of total arsenic and arsenic species: data for US children, adolescents, and adults | Excluded | No cognitive outcomes discussed |
| Vitamin B-6 Intake Is Modestly Associated with Arsenic Methylation in Uruguayan Children with Low-Level Arsenic Exposure | Excluded | No cognitive outcomes discussed |
| Bioaccessibility and speciation of arsenic in children's diets and health risk assessment of an endemic area in Bangladesh | Excluded | No cognitive outcomes discussed |
| Low level arsenic exposure, B-vitamins, and achievement among Uruguayan school children | Excluded | No cognitive outcomes discussed |
| Arsenite methyltransferase (AS3MT) polymorphisms and arsenic methylation in children in rural Bangladesh | Excluded | No cognitive outcomes discussed |
| Inorganic arsenic exposure and neuropsychological development of children of 4-5 years of age living in Spain | Excluded | No cognitive outcomes discussed |
| Prenatal exposure to arsenic and lung function in children from the New Hampshire Birth Cohort Study | Excluded | No cognitive outcomes discussed |
